# Getting closer to people: family planning provision by drug shops in Uganda

**DOI:** 10.9745/GHSP-D-14-00085

**Published:** 2014-12-02

**Authors:** Angela Akol, Dawn Chin-Quee, Patricia Wamala-Mucheri, Jane Harriet Namwebya, Sarah Jilani Mercer, John Stanback

**Affiliations:** a FHI 360 Uganda, Kampala, Uganda; b FHI 360, Durham, NC, USA; c FHI 360 Uganda, Kampala, Uganda, Now with the Clinton Health Access Initiative, Kampala, Uganda

## Abstract

**Background:**

Private-sector drug shops are often the first point of health care in sub-Saharan Africa. Training and supporting drug shop and pharmacy staff to provide a wide range of contraceptive methods and information is a promising high-impact practice for which more information is needed to fully document implementation experience and impact.

**Methods:**

Between September 2010 and March 2011, we trained 139 drug shop operators (DSOs) in 4 districts of Uganda to safely administer intramuscular DMPA (depot medroxyprogesterone acetate) contraceptive injections. In 2012, we approached 54 of these DSOs and interviewed a convenience sample of 585 of their family planning clients to assess clients' contraceptive use and perspectives on the quality of care and satisfaction with services. Finally, we compared service statistics from April to June 2011 from drug shops, community health workers (CHWs), and government clinics in 3 districts to determine the drug shop market share of family planning services.

**Results:**

Most drug shop family planning clients interviewed were women with low socioeconomic status. The large majority (89%) were continuing family planning users. DMPA was the preferred contraceptive. Almost half of the drug shop clients had switched from other providers, primarily from government health clinics, mostly as a result of more convenient locations, shorter waiting times, and fewer stock-outs in drug shops. All clients reported that the DSOs treated them respectfully, and 93% trusted the drug shop operator to maintain privacy. Three-quarters felt that drug shops offered affordable family planning services. Most of the DMPA clients (74%) were very satisfied with receiving their method from the drug shop and 98% intended to get the next injection from the drug shop. Between April and June 2011, clinics, CHWs, and drug shops in 3 districts delivered equivalent proportions of couple-years of protection, with drug shops leading marginally at 36%, followed by clinics (33%) and CHWs (31%).

**Conclusion:**

Drug shops can be a viable and convenient source of short-acting contraceptive methods, including DMPA, serving as a complement to government services. Family planning programs in Uganda and elsewhere should consider including drug shops in the network of community-based family planning providers.

## Abstract (precis)

Private drug shops can effectively provide contraceptive methods, especially injectables, complementing government services. Most drug shop clients in 4 peri-urban areas of Uganda were continuing users of DMPA; had switched from other providers, mainly government clinics, because the drug shops had fewer stock-outs and were more convenient (closer location, shorter waiting time, more flexible hours); and were satisfied with the quality of services. The drug shops provided a substantial part of the total market share for family planning services in their areas.

## INTRODUCTION

Drug shops are privately owned medicine outlets that are legally permitted to sell only nonprescription medications. Unlike pharmacies, drug shops are not required to employ trained pharmacists. Drug shops are often the first line of health care in sub-Saharan Africa,^1^ and, in countries such as Uganda that have high rates of maternal mortality and morbidity, they can play an important role in providing basic health care.^2^

Training and supporting drug shop and pharmacy staff to provide a wide range of contraceptive commodities and information is one of several promising high-impact practices in family planning,^3^ especially in light of evidence that paraprofessional health workers can safely administer injectable contraceptives in addition to providing oral contraceptive pills and condoms.^4^ However, more information is needed to fully document implementation experience and impact.

Private-sector drug shops are more readily found in underserved rural trading centers and peri-urban areas than in towns and cities, making them important players in increasing access to and use of family planning at the community level. In addition, as a private enterprise, drug shops are less likely than public-sector health facilities to suffer from commodity stock-outs and thus are potentially a sustainable source of contraceptive methods.^5^ Recognition of the utility of drug shops in providing short-acting contraceptive methods is growing in Africa.^6^^–^^8^ The recent introduction in Uganda and elsewhere of Sayana Press, a subcutaneous formulation of DMPA (depot medroxyprogesterone acetate), increases the prospects for non-clinic provision of DMPA and may catalyze policy action in favor of DMPA provision in drug shops.^9^^–^^11^

Drug shops are increasingly recognized as important providers of short-acting contraceptive methods.

In Uganda, where only 23% of currently married women use a modern contraceptive method but 34% of such women have unmet need for family planning,^12^ drug shops could play an important role in increasing access to family planning in hard-to-reach areas. A large proportion of family planning users (45%) obtain their methods from the private medical sector. Drug shops and pharmacies provide methods to 5.5% and 3.1% of users, respectively.^12^At 14% prevalence, intramuscular DMPA remains the method of choice for many women in Uganda.^12^

Drug shops in Uganda are regulated by the National Drug Authority (NDA), which mandates drug shops to sell only unrestricted or unclassified medicines. Within this policy, drug shops offer oral contraceptive pills and condoms.^13^ Administration of intramuscular DMPA is not included within the NDA policy, but in practice drug shops do provide DMPA.^2^ Drug shops depend on the commercial private sector to maintain their medicines supply chain, obtaining their supplies from large wholesale pharmacies who, in turn, procure contraceptives from social marketing or other commercial suppliers.

Under the STRIDES for Family Health project in Uganda, funded by the United States Agency for International Development (USAID), private drug shops were included, beginning in 2010, in efforts to expand the method mix of available contraceptives in Luwero and Nakasongola districts in central Uganda and in Mayuge and Bugiri districts in east-central Uganda. Uganda has 112 districts, with an average of 216,315 people in each district.^14^ By September 2011, 139 drug shops in the 4 selected districts had been identified and recruited by FHI 360, a STRIDES subcontractor, to provide family planning products and services. With the approval of the Ministry of Health (MOH), drug shop operators (DSOs) affiliated with these establishments were trained to counsel clients and administer DMPA injections.

In 2012, the PROGRESS (Program Research for Strengthening Services) project, a 5-year USAID-funded project implemented by FHI 360, carried out an enhanced evaluation to assess the contribution of drug shops to family planning service provision in the 4 selected districts of Uganda. Specifically, the evaluation estimated the market share of contraceptive method uptake of all methods provided by the drug shops and documented clients' level of satisfaction, perceptions of quality of care, counseling received, and intention to continue using drug shop family planning services.

## PROGRAM DESCRIPTION

### DSO Training

Between September 2010 and March 2011, we held 4 training workshops, one for each district, led by a national trainer closely assisted by the district officer in charge of family planning and reproductive health. In each workshop, we taught a maximum of 35 DSOs how to provide family planning methods, with an emphasis on DMPA, through several adult learning methodologies, including group discussions, demonstration and return demonstration (participants demonstrating back to the trainers what had just been demonstrated to them), and role play simulations. The training content included counseling clients to support informed choice, screening clients, procedures for ensuring infection prevention and injection safety, medical waste disposal, and referring clients for other methods or for complications. Each workshop was restricted to 2.5 days based on the assumption that the participants had some medical background and would already have some knowledge about family planning methods and their provision. Since the DSOs already knew how to provide intramuscular injections, we did not include a clinic practicum session. However, all participants were required to demonstrate competency with injections during classroom demonstrations.

The training was based on the following resource materials: *Family Planning: A Global Handbook for Providers* (www.fphandbook.org); a manual on initiating clients on DMPA developed by FHI 360; and family planning provider checklists. At the end of the training, we provided each participant with the following resource guides:

- Provision of Injectable Contraception Services Through Community-Based Distribution: Implementation Handbook
- Checklist for how to be reasonably sure a client is not pregnant
- Checklist for screening clients who want to initiate DMPA
- Checklist for screening clients who want to initiate combined oral contraceptives
- Family Planning Methods: A Flip Chart for Community Health Workers

(For full-text access to the first 4 resources, see the Community-Based Family Planning Toolkit at: https://www.k4health.org/toolkits/communitybasedfp/training; the last resource is available at: https://www.k4health.org/sites/default/files/FP_Flipchart_Community_Health_ Workers.pdf.)

DSOs completed a written pre- and post-training questionnaire. The average score at pretest was 28%, with a range of 20%–53%. At posttest, the average score was 71%, ranging from 40%–98%. Overall, the evaluations revealed that the participants had limited knowledge of the full range of contraceptive methods.

### Supportive Supervision of the DSOs

Following training, the DSOs returned to their shops and began to administer DMPA with supportive supervision from the district health management team and FHI 360 staff. The aim of supportive supervision was to enhance the DSOs' skills, collect data, and troubleshoot any problems. Supportive supervision was conducted through quarterly DSO meetings (at which time, DSOs were retrained by district staff if necessary), field visits from FHI 360 staff, and independent visits from the District Assistant Drug Inspector (DADI).

We later amended the supervision strategy to deploy the DADI with other district health management team members or FHI 360 staff to foster trust between the drug inspectors and drug shops. Prior to this project, the DADI's role was to enforce the NDA Policy by closing drug shops that were not in compliance with the law. In our experience, when the drug shop owners saw the DADI's vehicle in the village, the owners would close the drug shops, preventing the drug inspectors from conducting their supervision visits. Deploying the inspectors with other project staff helped change the perception of the DADI's role.

### Data Reporting

Data were collected on numbers of new and revisit family planning clients, clients counseled, and contraceptive products distributed. Initially, the DSOs were supposed to submit these data directly to the district's health management information system (HMIS). However, the DSOs had little motivation to submit the data since they did not receive public-sector commodities or transport or other allowances from the government. Therefore, FHI 360 collected and submitted the data to the districts on behalf of the DSOs, which helped ensure data accuracy prior to submission.

### Logistical Support to Drug Shops

To increase the incentive for drug shops to provide family planning, FHI 360 provided them with logistical support, comprising a storage cupboard for records and supplies, files and record books for recording family planning service data, counseling guides, and a job aid on the USAID family planning compliance requirements. We also labeled and branded the drug shop with the MOH family planning symbol, the name of the drug shop, and USAID and project logos. The drug shops provided socially marketed contraceptives and sold them at the retail price recommended by the social marketing agency. FHI 360 did not have any role in ensuring family planning commodity availability at the drug shop.

## METHODS

### DSO and Client Interviews

We randomly selected 54 drug shops from the list of 139 eligible shops to participate in the evaluation. Between July and August 2012, we approached the 54 drug shop owners and collected information on their age, sex, level of education, and highest professional qualifications achieved. We asked them to recruit, over a 6-week period, interested family planning clients (men and women) to participate in a cross-sectional survey. We aimed to recruit a minimum of 510 family planning clients, allowing us to estimate the client satisfaction rate with a 95% confidence interval within 5% precision. Drug shop operators were asked to keep track of client refusals to assess if the response rate dropped substantially (below 85%) to flag potential biases in the sample.

All clients recruited by the DSOs were interviewed, resulting in a sample of 585 clients (Table 1). Using a structured questionnaire, we interviewed these clients to assess their contraceptive method use and perspectives of, and satisfaction with, drug shop-provided family planning services. Information gathered by interviewers was entered and managed in Epi Data, version 3.1. We used clients' reports of contraceptive use to calculate the proportion of interviewees who were new to family planning and to DMPA use. A new family planning client was defined as any client who was using contraception for the first time ever. We performed frequencies and cross-tabulations to assess data on client satisfaction and perceptions of quality of care, as well as limited bivariate analyses to assess whether satisfaction, quality of care, counseling, or method/service point switching were associated with drug shop characteristics and settings.

**Table 1.** Distribution of Study Participants and Other Characteristics by District, Uganda

****	**Bugiri**	**Mayuge**	**Luwero**	**Nakasongola**	**Total**
Total population in 2002^a^	237,441	324,674	341,317	127,064	
No. of drug shop owners interviewed	16	14	12	12	54
No. (%) of drug shop family planning clients interviewed	181 (30.9)	168 (28.7)	112 (19.2)	124 (21.2)	585 (100.0)
No. of government clinics in the evaluation subcounties^b^	11	8	13	N/A	32
No. of community health workers in the evaluation subcounties^b^	30	30	30	N/A	90

a Data from the 2002 Uganda Population and Housing Census.^14^

b Family planning service statistics from government clinics and CHWs were used for the market share analysis.

### Analysis of Service Statistics

We also conducted a comparative retrospective review of service statistics from drug shops, community health workers (CHWs), and government clinics to determine the drug shop market share of family planning services. A list of all the clinics, drug shops, and CHWs in the project subcounties was obtained from the district health office. Information on family planning uptake from CHWs and drug shops was available from project monitoring records while information from clinics, including private clinics and hospitals, was obtained from HMIS records at the district health offices. Data from these 3 major sources of supply were entered into Excel to compute the family planning market share of drug shops. Market share calculations were based on data from 3 of the 4 selected districts that had the data disaggregated by subcounty, and the calculations spanned the 3 months (April, May, and June 2011) for which complete data from all sources were available. To calculate the drug shop market share, we computed the total number of couple years of protection (CYPs) delivered by drug shops as a proportion of all CYPs from all sources in subcounties that had participating drug shops.

## RESULTS

### Background Characteristics of DSOs and Clients

Of the 54 DSOs we approached, 76% were female. Their average age was 37 years, and the majority (92%) had a background in health care as a nurse, midwife, clinical officer, or nursing aide (data not shown).

The large majority of the drug shop operators we interviewed had a medical background.

The DSOs contacted 585 of their family planning clients, all of whom agreed to participate in the evaluation. The large majority (90%) of the clients were female, and most were of reproductive age with an average age of 28.8 years (range, 13–52 years) (Table 2). The clients had, on average, 3.4 children (range, 0–13), and 71% of them desired a child in the future. More than half of the clients were married (67%) while 16% were cohabiting, 10% were single, and 8% were separated, divorced, or widowed. One-quarter had attained secondary school education or higher, while 31% had not received any formal schooling.

**Table 2.** Background Characteristics of Drug Shop Family Planning Clients, N = 585

**Characteristics**	****
Sex, %	
Female	90.1
Male	9.9
Age,^a^ mean (range), y	28.8 (13.0–52.0)
Marital status, %	
Single	10.3
Married	66.5
Unmarried, living together	15.7
Separated/divorced/widowed	7.5
No. of children,^a^ mean (range)	3.4 (0.0–13.0)
Highest level of education completed, %	
Did not attend school	6.5
Kindergarten/nursery school	24.8
Primary	43.1
Secondary or higher	25.4
Missing	0.2
Works for money, %	
Yes	77.9
No	15.1
Missing	7.0
Type of work, %	
Running a shop/stall/business	44.3
Farming	24.1
Housewife	12.0
Other	12.6
Missing	7.0
Socioeconomic status, %	
Very low	30.3
Low	29.9
Medium	31.4
High	8.4
Desires a baby in the future, %	70.6

a Data are among 584 clients (missing data for 1 client).

The majority of clients (78%) worked for money; 44% ran small-scale businesses (eg, retail shops, food market stalls, secondhand clothing stores, and other types of retail business) and 24% practiced farming. Despite being involved in income-generating activities, 60% were categorized as low or very low socioeconomic status and only 8% were categorized as having a high socioeconomic status.

### Family Planning Use

**Figure 1.**
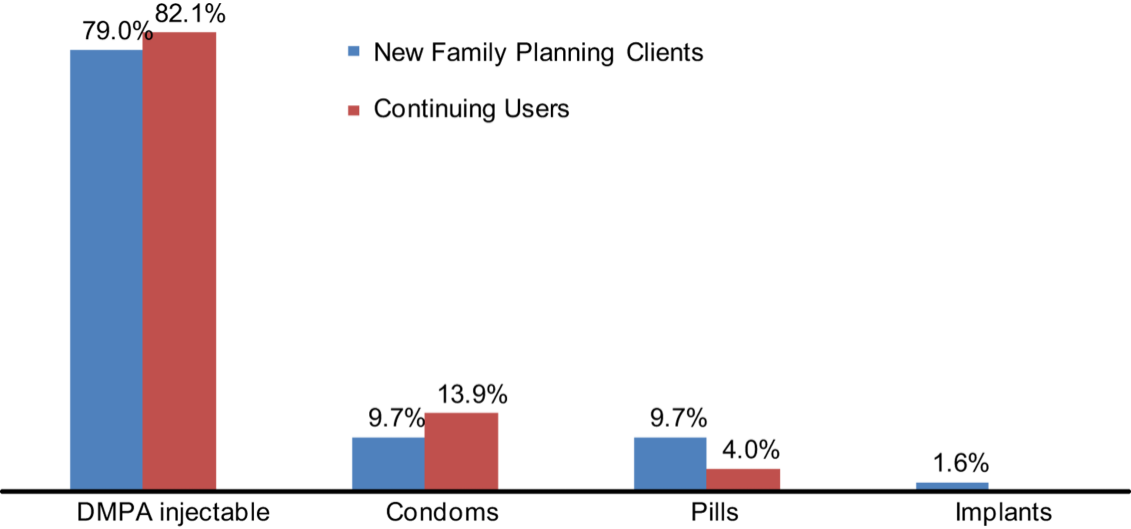
Client Method Choice at Drug Shops for New and Continuing Users, Selected Districts of Uganda, N = 585

About 11% of all interviewed drug shop clients were new family planning clients (Table 3). Most (79%) of the drug shop clients were using DMPA while 10% were using oral contraceptives and 11% were using condoms. The trend was similar among new family planning clients, with 79% choosing DMPA, and 9.7% each for condoms and pills (Figure 1).

**Table 3.** Contraceptive Methods Used by Drug Shop Family Planning Clients, N = 585

**Characteristic**	**Percent**
Method received at drug shop	
DMPA injectable	78.6
Condoms	10.9
Oral contraceptive pills	10.2
Implants^a^	< 1%
Ever use of family planning (FP)	
Used FP in the past, same method as current	60.3
Used FP in the past, different method from current	29.1
First-time user	10.6

a One client reported that she received an implant from a drug shop operator.

Most DMPA users were very satisfied with the method.

Among the 29% of clients who were method switchers (n = 170), the most commonly cited reason for switching was side effects (41%), followed by excessive or prolonged bleeding (24%) (Table 4). Nearly 1 client in every 5 cited issues related to logistics or adherence to the method as reasons for switching methods.

**Table 4.** Reasons for Switching Methods Among Family Planning Clients Reporting Use of a Different Method in the Past, n = 170

**Reasons^a^**	**Percent**
Side effects	41
Excessive/prolonged bleeding	24
Logistics/adherence	18
Couples’ discussion/preference	14
Other	12

a Total does not sum to 100% because clients could choose more than 1 reason.

Almost half of all drug shop clients (47%) had received their last contraceptive method supply elsewhere and were considered to have switched providers. Of those who had switched providers, the majority (66%) had switched from a government clinic/health center. Among those switching from a government location or a pharmacy, the most cited reason for switching providers was the convenient location of the drug shop (43%), while 12% mentioned that there was a shorter waiting time at the drug shop (Table 5). Other reasons mentioned with almost equal frequency were flexible hours of operation/better service (11%) and fewer stock-outs (10%) at the drug shop compared with clinics and health centers.

**Table 5.** Reasons for Switching to the Drug Shop Among Clients Who Switched From Pharmacies or Government Facilities, n = 184

**Reasons^a^**	**Percent**
Convenient location	43
Shorter wait time	12
Flexible hours of operation/better service or cost	11
Fewer stock-outs	10
Other	10
Missing	22

a Total does not sum to 100% because clients could choose more than 1 reason.

Ninety-two percent of the DMPA clients intended to get another injection. Among these, almost all (98%) mentioned the drug shop as the location of their next injection. Of the 10 clients who did not want to go to the drug shop, half cited money as the barrier (data not shown).

### Client Satisfaction and Perceptions of Quality of Care

Client reports of satisfaction and quality of care were positive. All clients (100%) reported that the DSOs treated them respectfully, and 93% trusted the DSO to maintain privacy (Table 6). About three-quarters felt that drug shop family planning services were affordable.

**Table 6.** Client Perceptions of Quality of Care and Client Satisfaction, N = 585

**Characteristic**	**Percent**
Friendliness of DSO	
Talked to client in a friendly way	89.1
Did not talk to client much	8.7
Talked to client in a unfriendly way	2.2
DSO treated client with respect	100.0
Trust the DSO will protect privacy	
Yes	93.3
No	1.2
Do not know	5.3
Missing	0.2
Feel family planning DSO services are affordable	
Yes	75.6
No^a^	21.7
Missing^a^	2.7
Will continue to go to DSO for family planning services	
Yes	94.0
No	5.5
Missing	0.5
Satisfied with the way the DSO provided the method	
Yes	99.0
No	1.0
Satisfied with DMPA^b^	
Very much satisfied	73.9
Somewhat satisfied	22.2
Not at all satisfied	3.3
Missing	0.6
Always go to same DSO for DMPA^b^	
Yes	90.0
No	9.8
Missing	0.2

Abbreviations: DSO, drug shop operator; DMPA, depot medroxyprogesterone acetate.

a Many of the clients with “no” or “missing” responses had received services for free.

b Data among DMPA users only (n = 460).

Among the surveyed DMPA users (n = 460, or 79% of the sample), about three-quarters were very satisfied with the method (Table 6). In addition, 96% would recommend the drug shop to a friend for family planning services, reflecting the overall high level of satisfaction with DMPA services from drug shops.

Client satisfaction with services was higher with female drug shop operators (74%) than with male (24%), although this difference was not statistically significant. Clients of female DSOs were significantly more likely to report the DSO had discussed side effects than clients of male DSOs (48% vs. 14%, respectively; *P* < .05) (Table 7). It is important to note, however, that there were many more female than male drug shop operators in the sample.

**Table 7.** Client Satisfaction and Reports of Counseling Received (%), by Sex of Drug Shop Operator (DSO),^a^ N = 585

****	**Services Received by:**		****
**Characteristic**	**Female DSOs**	**Male DSOs**	***P* Value**
Satisfaction with family planning services received at DSO			.54
Satisfied/somewhat satisfied	74.1	24.4	
Not at all satisfied	1.0	0.5	
DSO discussed:			
*Side Effects*	*48.2*	*13.5*	.*04*
Advantages	42.9	13.2	.48
Disadvantages	28.4	8.7	.75
Warning signs	42.1	12.7	.47
Would continue to go to DSO for family planning services	70.8	23.7	.74

*P* < .05 was considered statistically significant (shown in italics).

a The majority of the DSOs were women (42 female DSOs vs. 12 male DSOs).

### Drug Shops' Market Share of Family Planning Services

**Figure 2.**
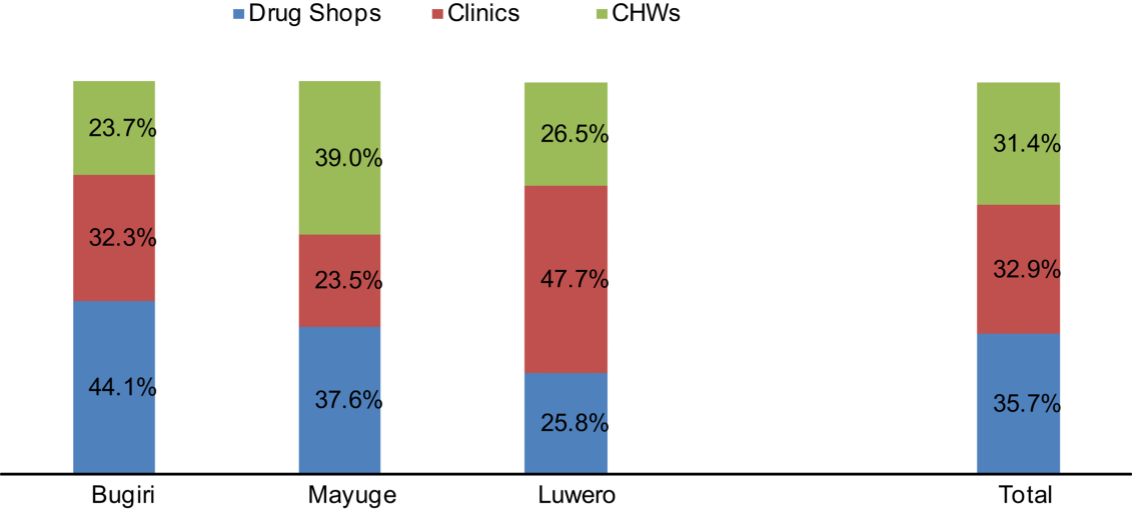
Market Share of Family Planning Services^a^ Provided by Clinics, Community Health Workers (CHWs), and Drug Shops in 3 Districts of Uganda, April–June 2011 ^a^ Measured by couple-years of protection delivered by each source.

Data from selected subcounties in 3 districts for April 2011 through June 2011 show that, overall, clinics, CHWs, and drug shops delivered equivalent proportions of CYPs to the community, with drug shops leading marginally at 36%, followed by clinics (33%) and CHWs (31%) (Figure 2). Variations existed within districts, with drug shops in Bugiri district enjoying the largest market share in that district (44%) and drug shops in Luwero district having the least market share (26%).

## DISCUSSION

Based on reports from the clients we interviewed, we found that drug shops serve more continuing than new family planning users, suggesting that drug shops may be better placed to resupply existing family planning users. We also found that almost half of all interviewed drug shop clients had received their previous method resupply elsewhere, mainly from government clinics. This switching behavior was attributed largely to more convenient locations, fewer stock-outs, and flexible hours and shorter waiting time at the drug shops than in clinics. These findings reflect nationwide statistics, which show that waiting time at government clinics is more than 6 times that at private clinics and that, at any given time, one-third of government health facilities are likely to face stock-outs of key health commodities, including DMPA.^15^

These findings suggest that drug shops, which are usually located in peri-urban areas where access to family planning and other health services is not as problematic as in rural areas, likely provide existing family planning users more convenience in resupplying their methods. Unmet need for family planning as a result of limited access to services, on the other hand, is largely a rural phenomenon. Another study in Uganda found that CHWs are more likely to serve *new* family planning users as a consequence of their placement in rural communities.^5^ Drug shops with trained providers can play an important role in peri-urban areas as an alternative source of contraceptive methods in cases where government clinics cannot meet client demand due to stock-outs or to geographical distance from users.

We also found that the quality of family planning services reported by drug shop clients was high. Clients unanimously agreed that the DSO treated them respectfully and almost all trusted the DSO to maintain their privacy. Perceived quality of services is considered to be a predictor of client satisfaction with services,^16^^,^^17^ which was indeed the case in our study. For DMPA, in particular, almost all clients indicated an intention to get their next injection from the drug shop. This is consistent with comparatively high satisfaction with private health care services found elsewhere.^18^^–^^20^ This high level of satisfaction, combined with the short waiting time, flexible hours, and convenient location as reasons for switching providers, suggests that drug shops are an acceptable provider of family planning, and particularly of DMPA resupply. In October 2014, the Ministry of Health in Uganda convened stakeholders to discuss the evidence on drug shops as a first step toward influencing policy change to allow DMPA provision in drug shops.

This proposition is reinforced by our finding that, drug shops, CHWs, and clinics deliver roughly equivalent amounts of CYPs to clients. However, there were differences in the drug shops' share of the family planning market among the 3 districts, with drug shops leading the market share in one district and having the least market share in another district. When we compared our market share findings with findings from a national assessment of the Uganda health system, we did not find the drug shop market share was related to the strength of the health systems in the 3 districts or to rural-urban differences in the 3 districts. This limits our ability to make inferences about the comparative strength of drug shops as a stakeholder in the general family planning market. Rather, this finding underscores the complementary role of the government and private sector in meeting people's family planning goals within the context of the total market approach to family planning.

The overall 36% market share of drug shops in the 3 districts included in our evaluation contrasts starkly with national data from the 2011 Uganda Demographic and Health Survey, which reports that only 3% of women get their methods from pharmacies and about 6% get them from drug shops.^12^ The reason for the disparity might be that DMPA provision in drug shops was a focus of the STRIDES project. Drug shop operators were trained in the safe provision of DMPA and provided with job aids and supportive supervision; thus, they were both empowered and motivated to counsel clients about DMPA and provide the method.

Finally, 3 drug shop clients of every 10 whom we interviewed did not want to have any more children. These are women for whom a more effective long-acting or permanent method of contraception might be suitable. This finding emphasizes the need for family planning programs to bring long-acting reversible contraception and permanent methods closer to clients. It also raises the prospect of drug shops as sources of information and referral for such methods.

### Strengths and Limitations

The quality and availability of data had an impact on our market share analysis. The market share analysis relied on HMIS records for family planning service provision data from clinics, but the HMIS did not always have such data. In addition, the market share analysis required data by subcounty, but in some cases, drug shop data were not disaggregated by subcounty and therefore could not be used in the analysis. Also, in some months (September 2010–March 2011), CHWs in some districts were not active, yielding no data. Thus, the market share analysis used data from only 3 months (April, May, and June 2011) for which complete data from all sources were available.

In addition, our sample of drug shop clients was a convenience sample drawn by asking the DSOs to invite their clients to participate in the survey. Therefore, the sample is not representative of all drug shop clients. In addition, there is a possibility that the DSOs invited only clients they considered to be satisfied clients, which would introduce a bias. However, since a large number of clients enrolled, the effect of this bias is probably not large.

## CONCLUSION

Drug shops can be a viable and convenient source of short-acting contraceptive methods, including DMPA injectables, particularly for continuing users but also for new family planning clients. Together with government services, drug shops and other private-sector providers offer complementary roles in meeting people's family planning needs and should be included in the network of community-based family planning providers in Uganda.

## Notes

**Peer Reviewed**

**First published online:** 2014 Nov 13

## References

[1] Wafula FN Miriti EM Goodman CA. Examining characteristics, knowledge and regulatory practices of specialized drug shops in Sub-Saharan Africa: a systematic review of the literature. BMC Health Serv Res. 2012;12(1): 223. 10.1186/1472-6963-12-223. 22838649 PMC3520114

[2] Stanback J Otterness C Bekiita M Nakayiza O Mbonye AK. Injected with controversy: sales and administration of injectable contraceptives in drug shops in Uganda. Int Perspect Sex Reprod Health. 2011;37(1): 24–29. 10.1363/3702411. 21478085

[3] High-Impact Practices in Family Planning (HIPs). High impact practices in family planning list. Washington (DC): US Agency for International Development; 2013 Available from: https://www.fphighimpactpractices.org/sites/fphips/files/hiplisteng.pdf

[4] Malarcher S Meirik O Lebetkin E Shah I Spieler J Stanback J. Provision of DMPA by community health workers: what the evidence shows. Contraception. 2011;83(6): 495–503. 10.1016/j.contraception.2010.08.013. 21570545

[5] Akol A Wamala P Krueger K Abbot A Scaling up community-based access to injectable contraceptives in Uganda: lessons learned from private- and public-sector implementation. Kampala, Uganda: Family Health International; 2009 Available from: https://www.k4health.org/toolkits/cba2i/scaling-community-based-access-injectable-contraceptives-uganda-lessons-learned

[6] Lebetkin E Orr T Dzasi K Keyes E Shelus V Mensah S. Injectable contraceptive sales at licensed chemical seller shops in Ghana: access and reported use in rural and periurban communities. Int Perspect Sex Reprod Health. 2014;40(1): 21–27. 10.1363/4002114. 24733058

[7] Chin-Quee D Ngadaya E Kahwa A Mwinyiheri T Otterness C Mfinanga S. Women's ability to self-screen for contraindications to combined oral contraceptive pills in Tanzanian drug shops. Int J Gynaecol Obstet. 2013;123(1): 37–41. 10.1016/j.ijgo.2013.04.024. 23859705

[8] Khan TU Malarcher S Ahmed S Sarker S Arevalo M The Blue Star Program: expanding access to injectable contraception through private sector outlets in Bangladesh. 2012 (Unpublished)

[9] Burke HM Mueller MP Perry B Packer C Bufumbo L Mbengue D. Observational study of the acceptability of Sayana® Press among intramuscular DMPA users in Uganda and Senegal. Contraception. 2014;89(5): 361–367. 10.1016/j.contraception.2014.01.022. 24631328

[10] Polis CB Nakigozi GF Nakawooya H Mondo G Makumbi F, Gray RH; Members of the Rakai Health Sciences Program Sayana Press study team. Preference for Sayana® Press versus intramuscular Depo-Provera among HIV-positive women in Rakai, Uganda: a randomized crossover trial. Contraception. 2014;89(5): 385–395. 10.1016/j.contraception.2013.11.008. 24332432

[11] Keith B Wood S Chapman C Alemu E. Perceptions of home and self-injection of Sayana® Press in Ethiopia: a qualitative study. Contraception. 2014;89(5): 379–384. 10.1016/j.contraception.2013.12.010. 24529492

[12] Uganda Bureau of Statistics (UBOS); ICF International. Uganda demographic and health survey 2011. Kampala (Uganda): UBOS; 2012. Co-published by ICF International. Available from: http://dhsprogram.com/pubs/pdf/FR264/FR264.pdf

[13] National Drug Policy and Authority Act 1993. Sect. 15. Available from: http://www.ulii.org/ug/legislation/consolidated-act/206

[14] Uganda Bureau of Statistics (UBOS). 2002 Uganda population and housing census: analytical report: population size and distribution. Kampala, Uganda: UBOS; 2006 Available from: http://www.ubos.org/onlinefiles/uploads/ubos/pdf%20documents/2002%20CensusPopnSizeGrowthAnalyticalReport.pdf

[15] Uganda Bureau of Statistics (UBOS). The Uganda national household survey 2012/13: socio economic findings. Kampala, Uganda: UBOS; 2013 Available from: www.ubos.org/onlinefiles/uploads/ubos/UNHS_12_13/UNHS-2012-131.zip

[16] Berendes S Heywood P Oliver S Garner P. Quality of private and public ambulatory health care in low and middle income countries: systematic review of comparative studies. PLoS Med. 2011;8(4): e1000433. 10.1371/journal.pmed.1000433. 21532746 PMC3075233

[17] Cho WH Lee H Kim C Lee S Choi KS. The impact of visit frequency on the relationship between service quality and outpatient satisfaction: a South Korean study. Health Serv Res. 2004;39(1): 13–34. 10.1111/j.1475-6773.2004.00213.x. 14965075 PMC1360992

[18] Tayue T Mirkuzie W Shimeles Ololo Determinants of patient satisfaction with outpatient health services at public and private hospitals in Addis Ababa, Ethiopia. Afr J Prim Health Care Fam Med. 2012;4(1): art. #384 10.4102/phcfm.v4i1.384

[19] Nketiah-Amponsah E Ulrich H Determinants of consumer satisfaction of health care in Ghana: does choice of health care provider matter? Glob J Health Sci. 2009;1(2): 50–61 10.5539/gjhs.v1n2p50

[20] Boller C Wyss K Mtasiwa D Tanner M. Quality and comparison of antenatal care in public and private providers in the United Republic of Tanzania. Bull World Health Organ. 2003;81(2): 116–122. 12751419 PMC2572401

